# Recalibrating Protection Factors Using Millisecond Hydrogen/Deuterium Exchange Mass Spectrometry

**DOI:** 10.1021/acs.analchem.4c03631

**Published:** 2025-01-29

**Authors:** Michele Stofella, Neeleema Seetaloo, Alexander N. St John, Emanuele Paci, Jonathan J. Phillips, Frank Sobott

**Affiliations:** † School of Molecular and Cellular Biology and Astbury Centre, University of Leeds, Leeds LS2 9JT, U.K.; ‡ Living Systems Institute, University of Exeter, Exeter EX4 4QD, U.K.; # Department of Biosciences, University of Exeter, Exeter EX4 4QD, U.K.; § Dipartimento di Fisica e Astronomia, Università di Bologna, Bologna 40127, Italy

## Abstract

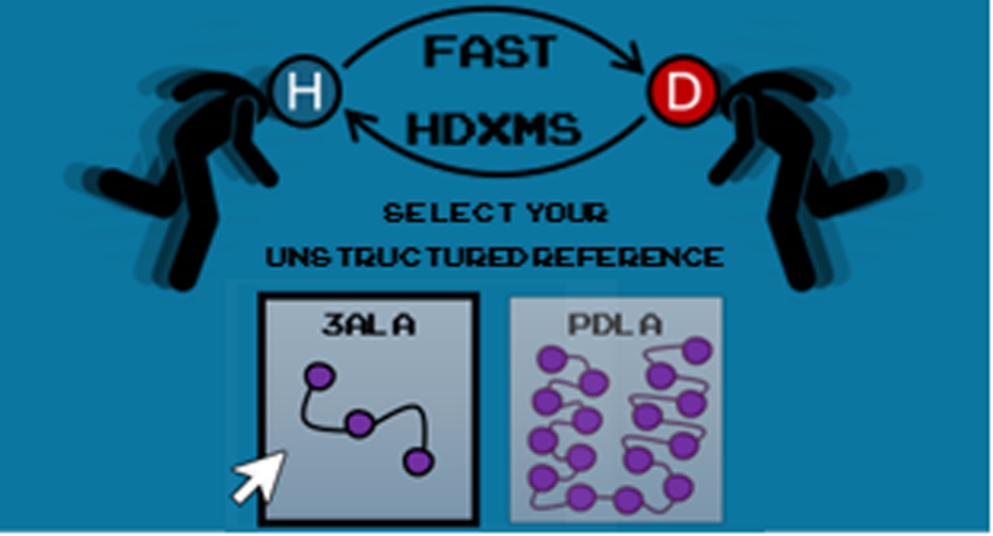

Hydrogen/deuterium exchange mass spectrometry (HDX-MS) is a powerful technique to interrogate protein structure and dynamics. With the ability to study almost any protein without a size limit, including intrinsically disordered ones, HDX-MS has shown fast growing importance as a complement to structural elucidation techniques. Current experiments compare two or more related conditions (sequences, interaction partners, excipients, conformational states, etc.) to determine statistically significant differences at a number of fixed time points and highlight areas of changed structural dynamics in the protein. The work presented here builds on the fundamental research performed in the early days of the technique and re-examines exchange rate calculations with the aim of establishing HDX-MS as an *absolute* and *quantitative*, rather than *relative* and *qualitative*, measurement. We performed millisecond HDX-MS experiments on a mixture of three unstructured peptides (angiotensin, bradykinin, and atrial natriuretic peptide amide rat) and compared experimental deuterium uptake curves with theoretical ones predicted using established exchange rate calculations. With poly-dl-alanine (PDLA) commonly used as a reference,^,^ we find that experimental rates are sometimes faster than theoretically possible, while they agree much better, and are never faster, with the fully unstructured trialanine peptide (3-Ala). Molecular dynamics (MD) simulations confirm the high helical propensity of the longer and partially structured PDLA peptides, which need as few as 15 residues to form a stable helix and are therefore not suitable as an unstructured reference. Reanalysis of previously published data by Weis et al. at 100 mM NaCl however still shows a discrepancy with predictions based on 3-Ala in the absence of salt, highlighting the need for a better understanding of salt effects on exchange rates. Such currently unquantifiable salt effects prevent us from proposing a comprehensive, universal calibration framework at the moment. Nevertheless, an accurate recalibration of intrinsic exchange rate calculations is crucial to enable kinetic modeling of the exchange process and to ultimately allow HDX-MS to move toward a direct link with atomistic structural models.

## Introduction

Protein structural dynamics and order–disorder transitions play an important, but often overlooked role in the human proteome, with approximately one-third of proteins being partly or fully disordered.^1^ Well-studied examples include the important tumor suppressor p53 and the abundant, Parkinson’s disease-related protein α-synuclein, which are known to undergo conformational transitions when interacting with DNA sequences and lipid bilayers, respectively. Moreover, the structural and functional behavior of intrinsically disordered proteins (IDPs) and their interactions in the crowded cellular environment often depend on biophysical parameters, such as pH, dielectric properties, ion concentrations, and macromolecular crowding.^2^ For example, IDPs can undergo conformational changes^3^ and even liquid–liquid phase separation^4^ at different salt concentrations. *In vivo*, proteins are solvated in diverse environments which can vary considerably within and between cells, e.g., with high intracellular concentrations of specific metal ions (up to 20 mM for Mg^2+^) and pH ranging from neutral in cytosol (6.8–7.2) to acidic conditions in endosomes and lysosomes (as low as 4.5).

While high-resolution structural techniques, such as X-ray crystallography and cryo-electron microscopy, can capture very detailed images of individual molecular states, the characterization of structural dynamics and intrinsic disorder under near-physiological conditions remain challenging. Over the past decade, hydrogen/deuterium exchange mass spectrometry (HDX-MS) has emerged as a powerful technique to fingerprint structural and dynamic properties of proteins^5−9^ in different solvent environments. HDX-MS utilizes the spontaneous exchange between amide backbone hydrogen atoms and deuterium in the solvent, which increases the mass of the protein and can be monitored by mass spectrometry to detect changes in the degree of hydrogen bonding per amino acid and determine the local structural dynamics. HDX-MS data retain information about the exchange of a protein at peptide-level resolution (5–10 amino acids). The same phenomenon (HDX) can be monitored at the level of the single residue using nuclear magnetic resonance (NMR) spectroscopy.^10^ Traditional HDX-NMR experiments probe exchange time scales of seconds/mins^11−13^ but can be pushed down to a submillisecond range.^14^ However, HDX-MS is more versatile, allowing the study of small molecules^15^ as well as MDa complexes,^16^ and it requires lower amounts of sample and is compatible with many buffers and solution conditions, therefore enabling studies in different chemical environments such as integral membrane proteins in lipid nanodiscs^17^ and biotherapeutics in formulations with added excipients.^18^ There is now fast growing interest in HDX-MS in the biopharma industry for the characterization of biotherapeutic molecules^19^ and epitope mapping.^20^ More recently, fast (millisecond) HDX-MS has emerged as a key technique^21−23^ to study weak (or fast cycling) binding interactions, allosteric effects, and dynamics of unstructured sequences in intrinsically disordered proteins (IDPs)^24^ that are associated with cancer and amyloid-related neurodegenerative diseases. It is desirable in such cases to map out conformational landscapes rather than just determining individual structures and to understand the factors (which might be environmental rather than intrinsic to the sequence itself) which govern transitions between different states, a formidable challenge which is well addressed by fast HDX-MS approaches aided by ensemble calculations using advanced computational methods.^25,26^

In HDX-MS, the backbone amide hydrogen-exchange rate is an important and highly sensitive measure of a protein’s structural dynamics.^27^ To accurately assess differences in the exchange kinetics, it is necessary to distinguish the impact of the chemical environment from that of the protein itself and its structural changes. The kinetics of exchange depends on the acidity/basicity of the respective amide protons which is determined by the sequence, i.e., the nature of each amino acid and its nearest neighbors,^28−31^ as well as on structural properties of the protein, which define the 3D microenvironment of an amino acid—mainly dictated by hydrogen bonding, electrostatics, and solvent accessibility.^32^ Exchange rates also depend on chemical properties of the solvent, which determine the mobility and activity of protons (H^+^/D^+^), which in turn is intimately linked with the availability of hydroxide ions as the actual catalytic agents initiating HDX (pH/pD, temperature, and ionic strength). While the effects of pH and temperature on OH^–^/OD^+^ activity can, in principle, be predicted by calculations, salt effects are usually not explicitly considered. An approach adopting an empirical buffer correction has been recently proposed, where a reporter peptide is used to detect differences in exchange caused by the introduction of additives in the buffer.^33^ A theoretical framework enabling the prediction of intrinsic exchange rates as a function of salt type and concentration is however lacking. Published empirical calculations of *intrinsic* exchange rates, which refer to the exchange rate of a residue in a completely unfolded chain, take some of these factors into account, and they are usually calibrated based on what is assumed to be a fully unstructured sequence. Current practice in HDX-MS relies on relative measurements of two or more states in direct comparison, and it interprets the differential exchange pattern at the peptide level *qualitatively*, based on statistical significance. This falls well short of what the method could, in principle, achieve with proper calibration. If true and correctly calibrated intrinsic rates were available, which take salt effects and accurate back-exchange estimations into account, “absolute” H/D exchange levels could be measured directly instead of differences between conditions. With such knowledge of *quantitatively* correct exchange rates, sets of protection factors could be determined, which are meaningful across separate experiments and different solvent environments. This would in turn also facilitate the use of such information in integrative structural modeling approaches, with the ultimate goal to combine HDX-MS and molecular dynamics (MD) for elucidation of protein structural ensembles. Here, we make some key steps toward this goal.

In this work, we performed millisecond HDX-MS experiments on a mixture of unstructured peptides to test the validity of the commonly used intrinsic exchange rate estimates provided by the Englander group,^28−31^ with the aim to determine an appropriate unstructured reference sequence. The assumption that the peptides are unstructured was validated by circular dichroism (CD) spectroscopy. The exchange of unstructured peptides is too fast to be detected in a “standard” HDX-MS instrument, where the minimum acquisition time is 20–30 s. The access to the millisecond time scale is proven here to be crucial to determine the correct intrinsic exchange rates and how they are influenced by the presence of salt. Our findings revealed that intrinsic exchange calculations are more accurate when a three-alanine peptide (3-Ala) reference is used instead of the standard poly-dl-alanine (PDLA), which retains some residual structure. We used MD simulations to confirm the high structural propensity of PDLA peptides, which had already been reported by several computational and experimental studies.^34−39^ The slower exchange rate of PDLA relative to 3-Ala had likely been overlooked so far, as it only becomes apparent at shorter time points (<20 s) than those typically used with “standard” HDX-MS experiments. Our results corroborate the fundamental validity of the established intrinsic exchange rate calculations when recalibrated using a proper unstructured reference such as 3-Ala. This is an essential step toward establishing HDX-MS as an *absolute* and *quantitatively correct* measurement, but for practical purposes, a more detailed understanding of salt effects will also be required in the future.

## Methods

### Theoretical Framework

In principle, HDX provides information at the resolution of a single amino acid. Indeed, the Linderstrøm-Lang model^27^ describes the exchange of each residue as a two-step process: the first guided by local fluctuations of the protein, and the second by the chemistry of the individual residue and the surrounding solvent. The observed rate of exchange
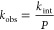
is defined as the ratio between the *intrinsic* exchange rate *k*_int_, representing the exchange rate of the amino acid in a completely unfolded chain, and the protection factor *P*, which can be interpreted as the “degree of protection” of the residue induced by the structure of the protein. Intrinsic exchange rates have been studied in the early days of HDX and their dependence on pH, temperature^40,41^ and side chains of the neighboring residues is widely accepted.^28−31^ On the other hand, the protection factor encodes structural properties of the residue within the protein:^6^ several microscopic models have been developed to link the structure of a protein with its protection factors, with satisfying outcomes.^32^ Retrieving a well-defined biophysical parameter, such as the protection factor, from HDX-MS experiments permits a correlation of the data with atomistic models of protein structure and dynamics obtained from complementary techniques, such as NMR, cryo-EM, or molecular dynamics (MD) simulations. Differential (i.e., relative and qualitative) HDX-MS data are extremely useful to locate the effect of a perturbation, but they make predictions of structural properties and correlation with other experiments rather challenging.^42^

Isolating the effect of chemistry (*k*_*int*_) is crucial to derive absolute structural information (P) from the observed data (*k*_*obs*_). Even in differential studies, omitting the deconvolution of these two effects can introduce a bias in the results or, worse, can lead to the wrong conclusions, mostly when studying conformational changes of proteins under different buffer conditions, e.g., when dealing with temperature- or pH-driven conformational changes.^43,44^ Consider that a minor change in pH can cause differences in the uptake curves that can be misclassified as significant structural changes. For example, we used the Englander intrinsic exchange rates to calculate that a difference in pH of 0.1 is sufficient to generate differences >0.5 Da in the uptake curve of an unstructured peptide with sequence AAAAAAAAAA at temperature 300 K (Supporting Figure 1).

One of the main challenges associated with deriving quantitative information (such as the absolute protection factors, rather than their relative differences) from HDX-MS data is the deconvolution of the peptide-level data provided by the experiment into single residue information.^45^ We have recently developed a computational method that exploits the additional information contained in the isotopic envelope to extract (most of) the protection factors of a protein from HDX-MS data^46^ and have shown that our estimates correlate well with NMR measurements.^47^ Our method relies on the accuracy of the intrinsic exchange rates, which we assumed to be correct and constant (for a given sequence at a fixed pH and temperature), following the empirical estimates developed by the Englander group.^28−31^ We decided to further challenge our assumption by studying the exchange of unstructured peptides, taking advantage of the recent developments in the acquisition of millisecond HDX-MS data.^21,24^

The intrinsic exchange rate estimates from the Englander group assume that the exchange rate of a residue in a completely unfolded structure depends mainly on three factors: (i) temperature, (ii) pD of the labeling buffer, and (iii) side chains of the neighboring residues.^28−31^ Additional factors, such as the reported dependency of the intrinsic exchange rate on salt concentration,^48^ are neglected. The temperature dependence follows the Arrhenius law, which is valid within the range of temperatures 0–60 °C provided that the protein structure remains stable, while it needs to be adjusted for higher temperatures.^44^ The dependence of the intrinsic exchange rate on the pD (pD = pH_read_ + 0.4) has a V-shaped curve with a minimum at pD 2.55 (this value is averaged over all amino acids). The dependence of the intrinsic rate on the neighboring side chains was empirically determined by studying all 20 naturally occurring amino acids with dipeptide models and comparing their exchange rates with polyalanine models.^30^ In their original paper,^28^ Bai et al. used NMR to determine the reference values for the left (L) and right (R) isomers of an alanine dipeptide (N-Ac-Ala-N′MA), for the internal NH of a blocked alanine tripeptide (N-Ac-Ala-Ala-Ala-N’MA) and for a racemic poly-dl-alanine (PDLA) with degree of polymerization 28 (which represented the average length of the polypeptides). The reference rates were measured in the presence of 0.5 M KCl and then extrapolated to “low salt concentration”.^28^ In a follow-up study, the Englander group adjusted the reference values for PDLA (at low salt concentration) by a factor of 1.35, after comparing the exchange of PDLA peptides of different lengths with apolipoprotein C3, which was assumed to be completely unstructured.^31^ However, several studies have criticized the validity of these calculations because they could not match the predictions with experimental data: the experimental uptake was found to be faster than the predicted one, which for a fully unstructured reference should be the fastest exchange possible on that amino acid (at a fixed pH and temperature).^23,49−52^

### Materials

Deuterium oxide (99.9% D_2_O) was purchased from Goss Scientific (catalog number: DLM-4). The peptide mixture (Supporting Table 1) contained three peptides (10 μM each): angiotensin (A9202, Sigma-Aldrich), bradykinin (90834, Sigma-Aldrich), and ANP (atrial natriuretic peptide) amide rat (SCP0022, Sigma-Aldrich). We performed circular dichroism (CD) spectroscopy experiments to validate the assumption that the peptides are completely unfolded (Supporting Figure 2).

### Hydrogen/Deuterium Exchange Experiments

Hydrogen–deuterium exchange (HDX) was performed using a fully automated, millisecond HDX labeling and online quench-flow instrument, ms2min (Applied Photophysics, U.K.),^21,24^ connected to an HDX manager (Waters). The peptide mixture (Supporting Table 1) in the equilibrium buffer (20 mM Tris, pH 7.40) was delivered into the labeling mixer and diluted 20-fold with labeling buffer (20 mM Tris, pH_read_ 7.00) at 20°C, initiating HDX at 95% deuteration. The labeling times depended on the varying length of mixing loops in the sample chamber and the flow rate of the carrier buffer. The peptides were labeled for a range of times from 50 ms to 5 min. Immediately postlabeling, the sample was mixed 1:1 with quench buffer (100 mM Tris, pH = 2.55 for the mixture of equilibration and quench buffer) in the quench mixer to minimize any further exchange. The sample was loaded into the HPLC injection loop of the ms2min and sent to the HDX manager. The peptides were trapped on a VanGuard 2.1 mm × 5 mm ACQUITY BEH C18 column (Waters) for 3 min at 7000–9000 psi and separated on a 1 mm × 100 mm ACQUITY BEH 1.7 μm C18 column (Waters) with a 4 min linear gradient of acetonitrile (15–40%) supplemented with 0.1% formic acid. The eluted peptides were analyzed on a Synapt G2-Si mass spectrometer (Waters, Wilmslow, U.K.). An MS-only method with a low collisional activation energy was used: fragmentation was not needed as we wanted to study the exchange of intact peptides with known sequence. Up to four technical replicates were collected. Deuterium incorporation into the peptides was measured in DynamX 3.0 (Waters).

### Data Processing and Analysis

The evolution of the isotopic envelopes of the three peptides was monitored as a function of time. We calculated the experimental fractional deuterium uptake as
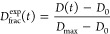
1where *D*(*t*) is the centroid (intensity-weighted average) of the isotopic envelope of the peptide at time *t*, *D*_0_ is the centroid of the fully protonated envelope (no exchange), and *D*_max_ is calculated as the centroid of the maximally deuterated envelope (after 5 min labeling when the uptake reached a plateau). The experimental fractional uptake was averaged over the replicates available, and the error associated with experimental measurements was the pooled standard deviation (Supporting Figure 3).

The theoretical fractional uptake was calculated using a sum of exponentials:
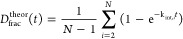
2where *N* is the number of exchangeable residues in the peptide (prolines are excluded) and *k*_int,i_ is the intrinsic exchange rate of residue *i*. Note that the first residue is excluded from the sum, because of the lack of an amide at the *N* terminus. To calculate the intrinsic exchange rate, we used a Python script (available at https://github.com/pacilab/exPfact)^46^ adapted from the spreadsheet of the Englander Lab (https://hx2.med.upenn.edu/download.html). The intrinsic exchange rate of one residue can be predicted from the knowledge of temperature, pH, and side chains of the neighboring residues^28−31^ and was calculated using either polyalanine (PDLA) or the internal amide hydrogen of an alanine tripeptide (3-Ala) as references (Table 1).

**Table 1:** Hydrogen/Deuterium (HD) and Deuterium/Hydrogen (DH) Exchange Rate Constants for Alanine-Based Reference Molecules at 293 K^a^

reference	exchange	log *k*_A_ (M^–1^·min^–1^)	log *k*_B_ (M^–1^·min^–1^)	log *k*_W_ (min^–1^)
3-Ala	HD	2.04	10.36	–1.5
PDLA	HD	1.62	10.05	–1.5
PDLA	DH	1.40	10.00	–1.6

a The values were empirically determined in previous work^28−31^ by fitting experimental curves depicting the V-shaped dependence of the exchange rate of these reference molecules on the pD with the equation: *k*_ex_ = *k*_A_10^–pD^ + *k*_B_10^(pD–p*k*_D_)^ + *k*_W_. Reference parameters for PDLA are available for forward (H to D) and reverse (D to H) exchange; the reference parameters for 3-Ala are available for forward exchange only.

The agreement between experimental (eq 1) and predicted (eq 2) fractional uptake was evaluated using the sum-of-squared residuals (SSR) over the *J* time points available:
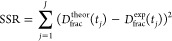
3

A pooled standard deviation was used (rather than individual errors for different measurements) in order to achieve a more accurate overall variability when dealing with multiple small samples from related populations.

### Molecular Dynamics Simulations

Racemic polyalanine peptides (50% d-alanine, 50% l-alanine, alternated) were constructed in PyMOL version 2.5.2. Acetyl and amide caps were added to neutralize each terminal charge. d-alanine residues were introduced manually by exchanging the Hα and methyl group (containing the Cβ, Hβ1, Hβ2, and Hβ3 atoms). Parameter and topology files were obtained using Tleap,^53^ the ff19SB^54^ and TIP3P force fields for peptide and water molecules, respectively. Each peptide was solvated with a water box that extends at least 12.0 Å away from any peptide atom. Potassium and chloride ions were added to obtain a concentration of 0.5 M KCl.^55^ Hydrogen mass repartitioning was carried out with ParmEd^53^ to facilitate a time-step of 4 fs. Each system was minimized using AMBER with 2500 steps of the steepest descent followed by 2500 steps of the conjugate gradient algorithm or until convergence. A harmonic restraint was applied to peptide atoms during minimization, and a 9 Å nonbonded interaction cutoff distance was used. After minimization, equilibration molecular dynamics (MD) was carried out using PMEMD^56^ with a 1 fs time-step in the NVT ensemble, during which the temperature was slowly increased from 0 to 293 K for 125 ps using Langevin dynamics with a collision frequency of 1 ps^–1^. All bonds apart from those containing hydrogen were constrained using the SHAKE algorithm.^57^ Production runs followed equilibration dynamics for 200 ns using an increased time-step of 4 fs in the NPT ensemble, where a 1 atm pressure was maintained using a Monte Carlo barostat. Snapshots were saved every 100 ps during the production runs and secondary structure propensity was calculated as an average over the snapshots using the DSSP algorithm.^58^

## Results

### Intrinsic Exchange Rate Predictions Are More Accurate When 3-Ala Is Used as a Reference

**Figure 1:**
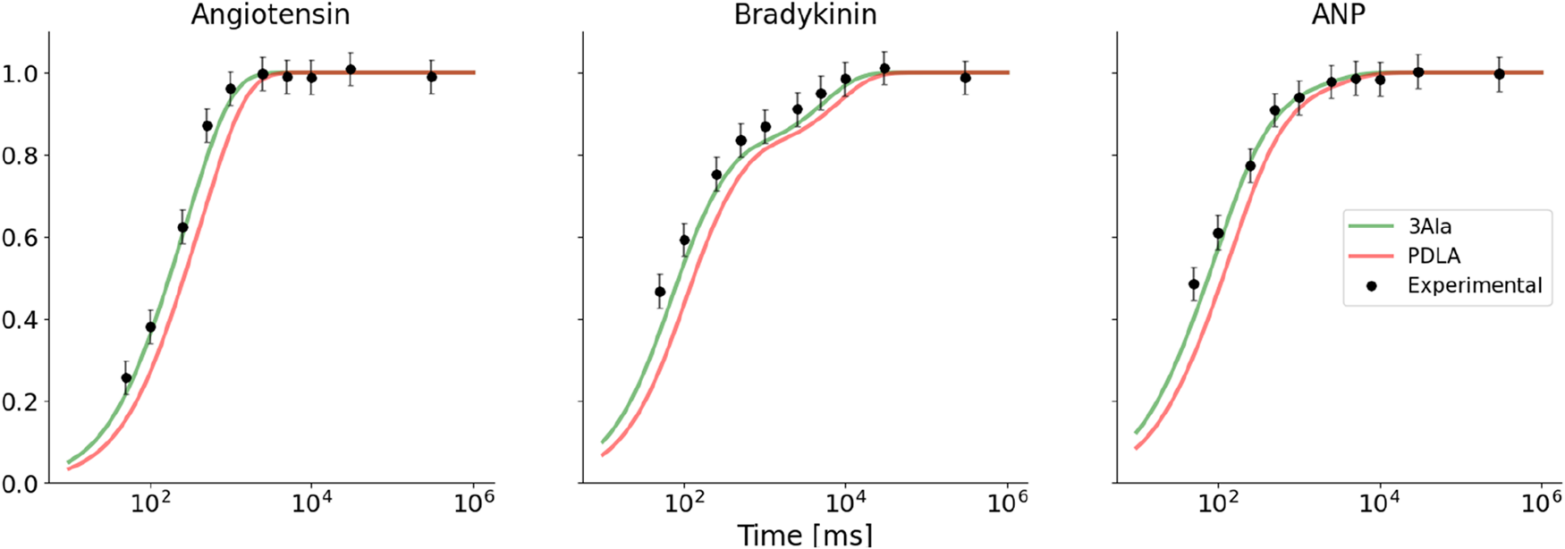
Hydrogen–deuterium exchange of angiotensin, bradykinin, and ANP. The experimental fractional uptake data (black) are compared with theoretical deuterium uptake calculated using the intrinsic exchange rate calculations from Englander using 3-Ala (green) or PDLA (red) as reference. The error associated with the experimental measurements is the pooled standard deviation.

Experimental data showing the fractional uptake (eq 1) of angiotensin, bradykinin, and ANP, assumed to be unstructured following CD experiments (Supporting Figure 2), in the absence of salt are shown in Figure 1. We predicted the fractional uptake (eq 2) of the peptides using the intrinsic exchange rate calculations by Englander,^28−31^ using either 3-Ala or PDLA as reference. The monoisotopic mass detected for ANP (Supporting Table 1) reflects the formation of a disulfide bond between residues C_4_ and C_15_, so the parameters for cystine (and not reduced cysteine) were used in the intrinsic exchange rate calculations. To reproduce the uptake of bradykinin, we had to make some assumptions about the configuration of the prolines. Prolines do not exchange because they do not have an amide hydrogen, but their cis/trans isomerization affects the exchange rate constants of neighboring residues. Indeed, different parameters are tabulated in the intrinsic exchange rate calculations for trans or cis proline. The deuterium uptake curves predicted by alternative bradykinin conformations are shown in Supporting Figure 4. Ion mobility studies have shown that the most probable conformation corresponds to trans-Pro_2_, trans-Pro_3_, and cis-Pro_7_ (Supporting Table 2).^59^ The deuterium uptake predicted for this conformation is shown in Figure 1.

The predictions were compared with experimental data, and the agreement was evaluated using the sum-of-squared residuals (SSR, eq 3). The SSR was 0.092 for angiotensin, 0.096 for trans–trans–cis bradykinin and 0.067 for ANP when PDLA was used as reference. Switching the reference from PDLA to 3-Ala reduced the SSR by approximately 1 order of magnitude: 0.011 for angiotensin, 0.022 for bradykinin, and 0.010 for ANP, values compatible with the pooled standard deviation σ_pooled_ = 0.041. Our experimental measurements showed a faster exchange than the theoretical exchange of fully unstructured peptides when PDLA was used as reference, while they matched the predictions much better when 3-Ala was used as reference.

### 3-Ala Rather Than PDLA Is a Suitable Unstructured Reference

**Figure 2:**
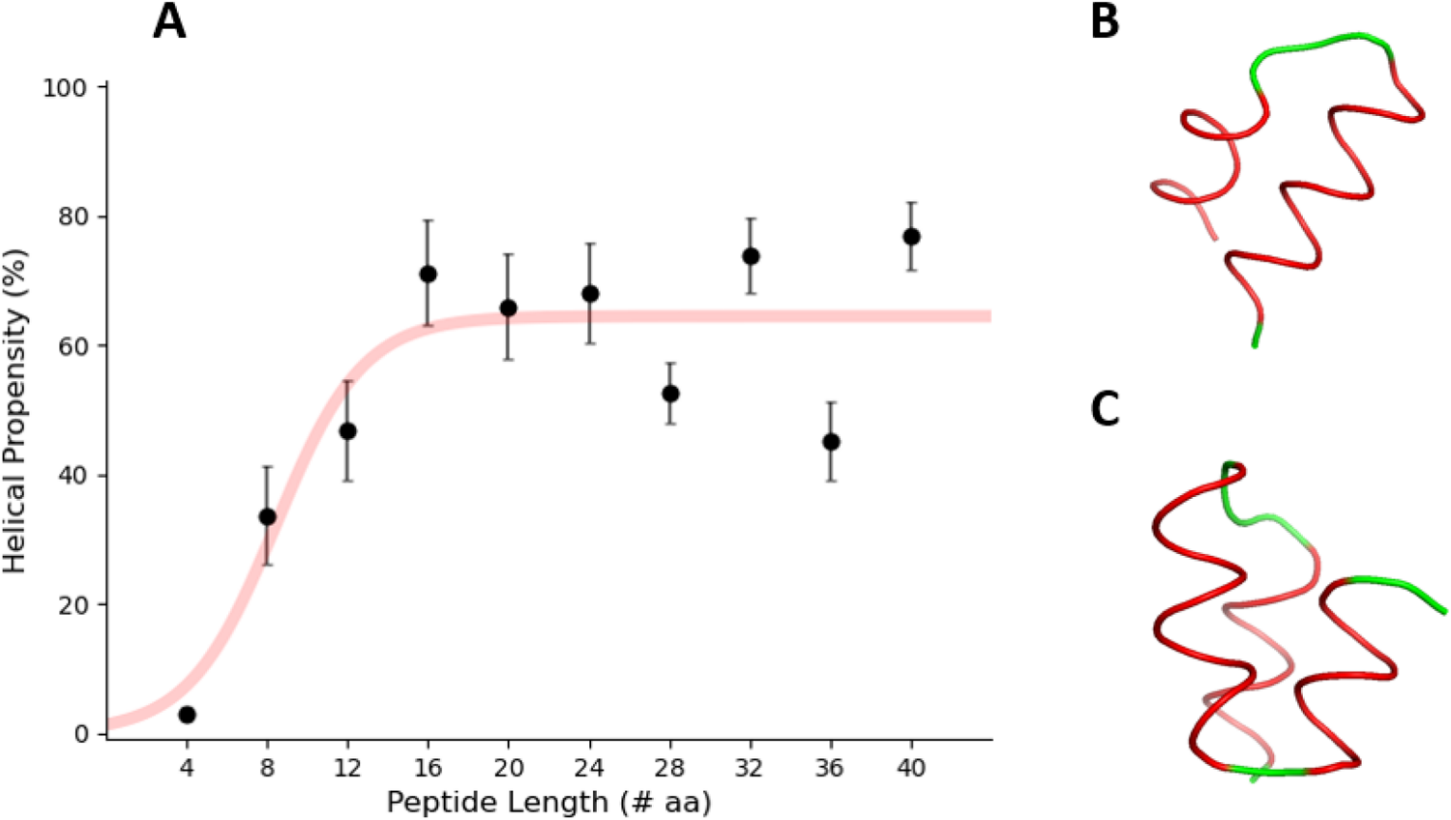
Structural propensity of PDLA peptides of increasing length from molecular dynamics simulations. (A) The helical propensity, calculated by using the DSSP algorithm and averaged over the amino acids of the peptide, is shown as a function of the peptide length. The error bars associated with the helical propensity are the standard deviations. Snapshots were taken every 100 ps of the simulation. (B, C) Snapshots of a double helical bundle from the simulations of PDLA with 24 residues (B) and a triple helical bundle for PDLA with 32 residues (C).

Molecular dynamics simulations of racemic PDLA (50% l-alanine, 50% d-alanine, alternated) highlighted its structural propensity. To replicate the experimental conditions used by Bai et al.,^28^ we simulated the behavior of PDLA in the presence of 0.5 M KCl. We performed simulations for PDLA peptides of increasing lengths (from 4 to 40 amino acids, with steps of 4) and measured the secondary structure propensity using the DSSP algorithm.^58^ The average helical propensity per amino acid over the simulation time is reported in Supporting Figure 5. The results in Figure 2 show the helical propensity averaged over the amino acids as a function of peptide length. The simulations highlight that a few alanine residues are sufficient to form helical conformations, with double (Figure 2B) or triple helical bundles (Figure 2C) forming at increasing peptide lengths. These results confirm our hypothesis, already supported by several experimental and computational findings,^34−39^ that PDLA is not a suitable unstructured reference.

**Figure 3:**
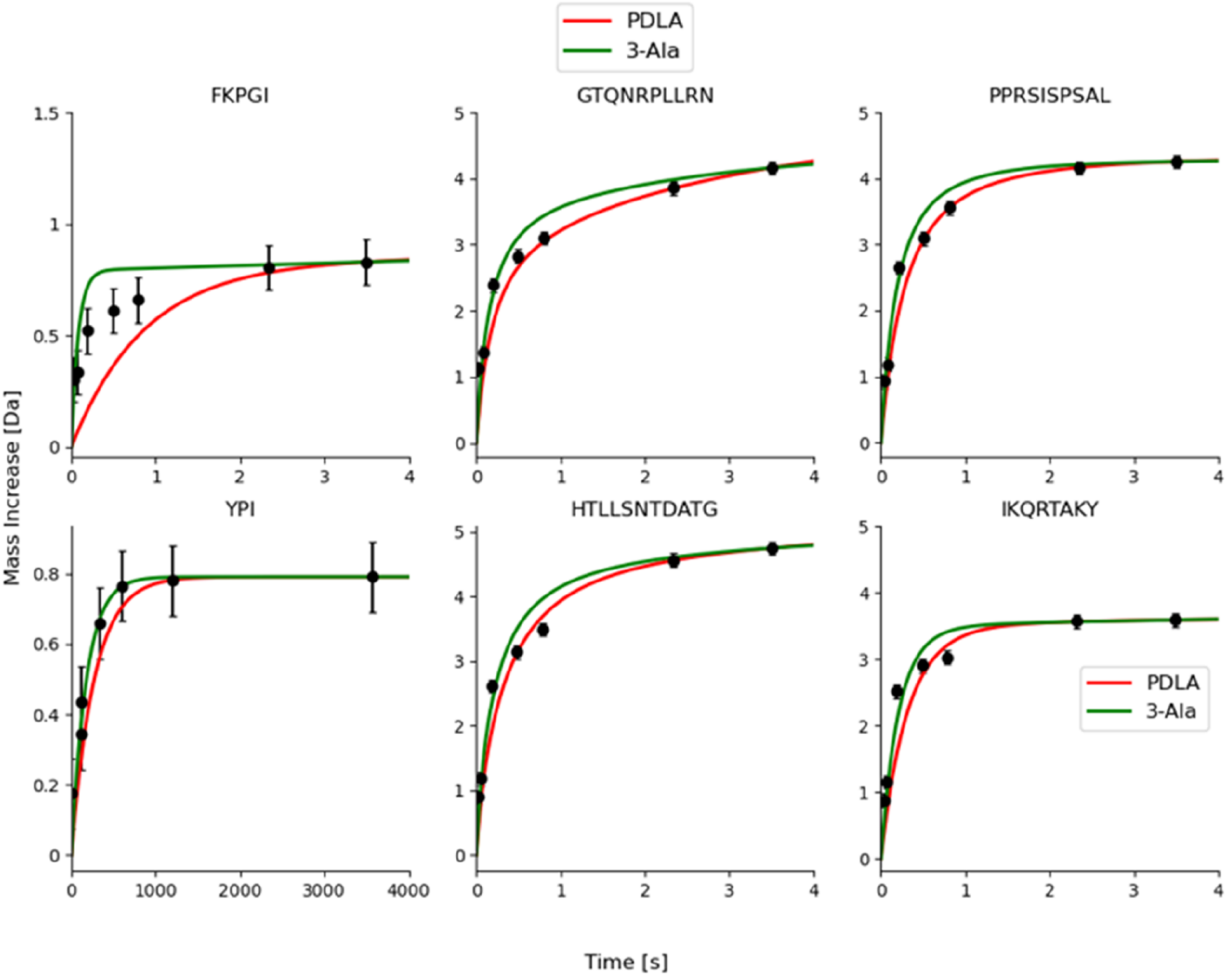
Experimental HD exchange data of unstructured peptides (black) previously published by Al-Naqshabandi and Weis^23^ is compared with the deuterium uptake calculated by us, using PDLA (red) or 3-Ala (green) as reference and accounting for the corrections published in ref (31). Data points were extracted from figures published in ref (23) using a plot digitizer, and a default error of ±0.1 Da was assigned to experimental measurements. Mass increase is shown on a linear time scale instead of fractional uptake on a logarithmic time scale to facilitate direct comparison with the original paper. The cis-Pro assumption is required for the peptide FKPGI.

Using PDLA as reference, several studies have observed that the intrinsic exchange rate calculations predicted an exchange *slower* than the experimental exchange of unstructured peptides or proteins.^49−52^ This is in principle not possible because intrinsic exchange rates should describe the exchange of a fully unstructured peptide, i.e., the fastest exchange possible for a given amino acid sequence at a given temperature and pH. However, the calculations used in these studies (i) used PDLA as reference instead of 3-Ala and (ii) did not account for minor corrections in the reference parameters that were introduced later.^31^ For example, Al-Naqshabandi and Weis showed that intrinsic exchange rate calculations were not able to reproduce the experimental curves for model peptides or unstructured proteolytic peptides of intrinsically disordered proteins.^23^ They found that observed exchange rates appear to be sometimes faster than the theoretically possible (fully deprotected) maximum, which prompted us to hypothesize that the currently used calibration reference (PDLA) could be the cause. We compared the experimental exchange of a subset of these peptides with the deuterium uptake calculated using either PDLA or 3-Ala as a reference (Figure 3). The exchange kinetics predicted using PDLA as reference (red curve) were either equal or *slower* than the experimental exchange, even after introducing the corrections implemented by Nguyen et al.^31^ for the intrinsic exchange rate calculations. When we predicted the exchange using 3-Ala as a reference (green curve), the predicted mass increase was either equal or *faster* than the experimental data. Nevertheless, unlike in Figure 1, here the 3-Ala based prediction appears at first sight to agree poorly with the experiment, which we ascribe to confounding salt effects (see the next section).

### Intrinsic Exchange Rate Depends on the Ionic Strength of the Buffer

**Figure 4:**
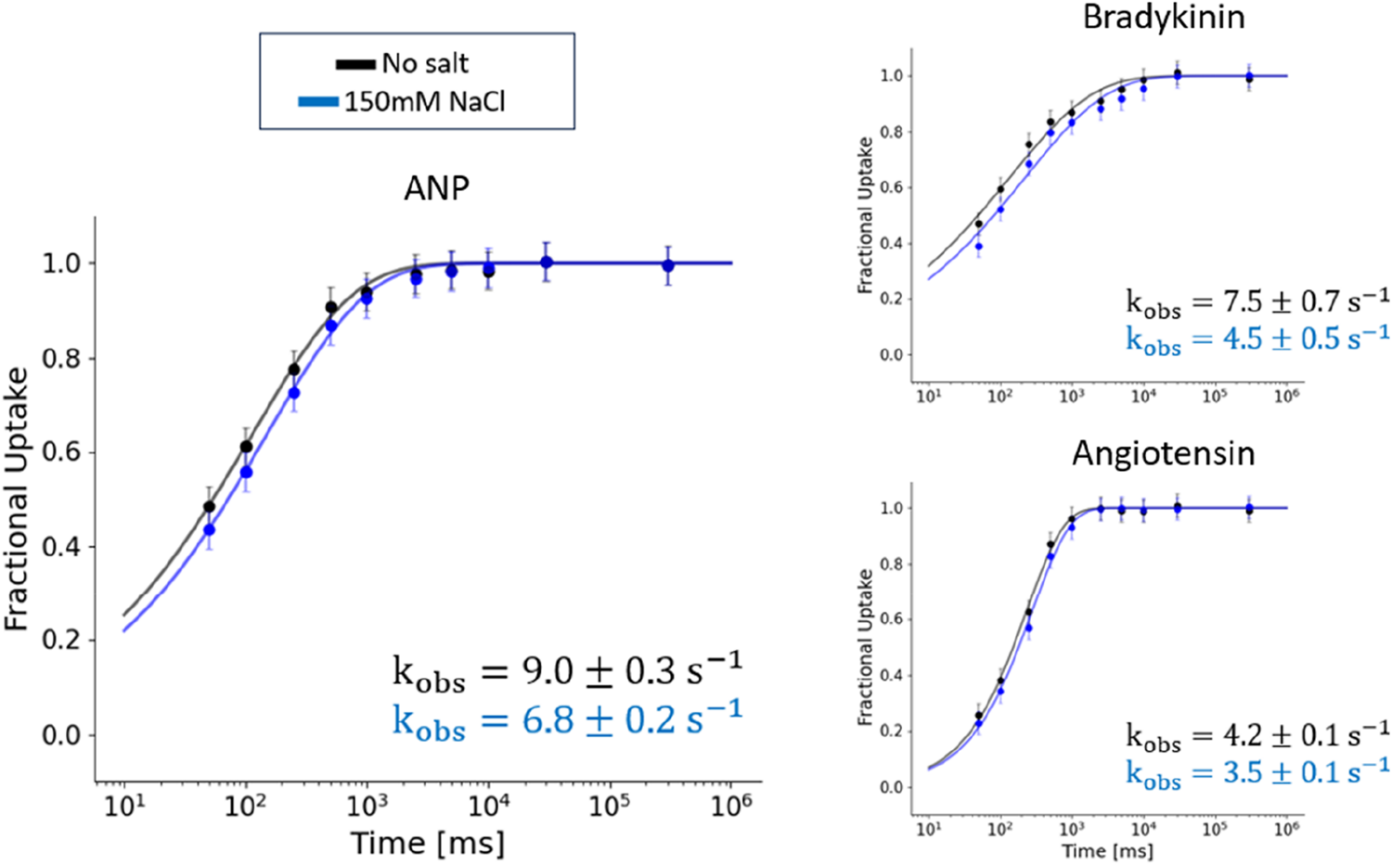
Fractional uptake of angiotensin, bradykinin, and ANP in the absence (black) or presence of 150 mM NaCl (blue). The error associated with experimental measurements is the pooled standard deviation. Experimental data are fitted with a stretched exponential model, and the observed rates *k*_obs_ are reported.

The results in Figure 3 show that the predicted exchange (with 3-Ala as the correct reference, green) is always faster than the experimental uptake for these unstructured peptides. But why is the observed exchange for these data *slower* than the prediction, and does not appear to match well in contrast to Figure 1? We suggest that this can be explained by the presence of salt in the buffer used in these experiments (100 mM NaCl), whereas our own measurements reported in Figure 1 were done without any salt.^23^ Importantly, all theoretical rate predictions (with PDLA or 3-Ala) are based on no-salt conditions, meaning that the data in Figure 1 are salt-matched, while those in Figure 3 are not. The dependence of the intrinsic exchange rate on the salt type and concentration had already been reported.^48^ Bai et al. measured the *k*_int_ for all amino acids in the presence of 0.5 M KCl “to shield possible charge effects”^28^ and they extrapolated the values at “low salt concentration” by comparing their results with data previously published in the absence of salt.^29^ Only the parameters at low salt concentration were reported in the well-known spreadsheet used for intrinsic exchange rate calculations (https://hx2.med.upenn.edu/download.html) and used in the follow-up study by Nguyen et al.^31^ Moreover, in a recent paper Toth et al. proposed the use of a reporter peptide to experimentally evaluate the effect of different buffer conditions on the exchange.^33^ We also conducted our own experiments to confirm here this additional dependence of the intrinsic exchange rate on the concentration (i.e., the ionic strength) of the salts in the labeling buffer. We measured the exchange of angiotensin, bradykinin, and ANP in the presence of 150 mM NaCl (Figure 4). The experimental curves were fitted with a stretched exponential (*D*_frac_ = 1 – e^–*k*_obs_*t^q^*^) and showed that the introduction of salt in the buffers slows down the exchange (subtly, yet significantly) of the peptides, as was expected. This salt effect does, at least qualitatively, explain the discrepancy between 3-Ala based predictions and the experimental data which we reanalyzed in Figure 3.

## Discussion

HDX-MS measures an observable (deuterium incorporation) that is related to structural properties of the protein,^32,60,61^ and it has been proven powerful in deriving data-driven structural models in combination with reweighting techniques and computational modeling.^25,62,63^ To achieve this goal, it is crucial to separate the effect of the buffer on the exchange pattern from that of the structure of the protein. Here, we take key steps toward establishing a framework for a quantitative, “absolute” analysis of H/D exchange rates, which would ultimately enable a direct connection between the HDX-MS data set and the 3D protein model or ensemble. We identify the fully unstructured trialanine peptide (3-Ala) as a more suitable reference peptide for intrinsic exchange rate calculations than the commonly used racemic poly-dl-alanine peptide (PDLA; Figures 1 and 3). Both references were already published in the original paper by Bai et al.,^28^ but only PDLA has been used in later studies. We show that PDLA is partially structured (Figure 2) and therefore cannot be used as a fully unprotected reference. After switching to a suitable reference (3-Ala), the observed exchange is either compatible with or slower (but never faster) than predicted, for the peptides in our mixture and for a set of disordered peptides previously published.^23^ We further confirm that intrinsic exchange rates have an additional dependence on salt concentration, which has so far not been considered in the theoretical *k*_int_ predictions. The addition of salts slows the exchange, which can explain the remaining discrepancy that the experimental curves sometimes show *slower* (and not equal) exchange than predicted (Figure 3). Nevertheless, a comprehensive and quantitative understanding of salt and buffer effects on exchange rates is currently missing. Within such a framework, which we are currently working to establish, we predict that 3-Ala can be used as a universal calibrant across all time scales and salt conditions, which is important not just for accurate protection factor calculations but also when comparing protein conformations in different chemical environments, e.g., excipients or under phase-separating conditions.

PDLA cannot be used as a fully unstructured reference because it displays slower exchange than 3-Ala as well as some other peptides, and we show that this is due to it having a high helical propensity above a length of ca. 10–15 amino acids (Figure 2). PDLA has been used as a standard at different temperatures and different pH in a publication by Linderstrøm-Lang et al. more than 65 years ago.^64^ At the time, the authors remarked that “the slow exchange may be explained by a stabilization of the helix due to internal nonpolar bonds between the methyl groups of the side chains”. Hence, we are actually not surprised that PDLA turned out to be unsuitable for exchange rate calculations as it retains some protection despite its mixed D/L stereochemistry. Even Bai et al. in their original paper stated that “the NH and CαH resonances of the PDLA sample showed some substructure, apparently intrinsic to interactions of the D and L residues”.^28^ The structural propensity of PDLA was also reported by Frushour and Koenig using Raman spectroscopy: “When PDLA is dissolved in water, the spectra suggest that short α-helical segments are formed upon dissolution”.^65^ PDLA has probably been preferred to 3-Ala in the context of HDX-MS experiments because the exchange of 3-Ala is too fast to be detected with a “standard”, i.e., manual or robotic workflow which is generally able to detect time points at or above 20–30 s. This highlights the importance of the millisecond time scale for fundamental studies of HDX. As a side technical note, PDLA peptides exhibit high hydrophobicity, making their purification quite challenging. This reinforces the argument that PDLA is not a good reference model.

For “standard” HDX experiments with time points >20 s, the difference between using either calibrant might appear insignificant, particularly when buffers are not salt-matched. In our example (Figure 3; red/green curves vs black data), the error made by neglecting salt dependence can be equal or even bigger than when using the wrong reference compound. The two confounding factors in these data illustrate that use of an appropriate reference and correct salt-matching are both important; yet, they are independent of each other, and correct calibrations should be used with all buffer conditions. It is important to emphasize that salt can of course affect the higher-order structure of the analyte, but the unstructured peptides used here for reference (3-Ala) and testing (Figures 1 and 3) are assumed to be salt-independent. Rather than the analyte itself, the observed changes in H/D exchange rates are believed to be due to salt effects on the higher-order structure of surrounding water and its corresponding proton (and correlated hydroxyl ion) mobility (activity), similar to how the pH and temperature determine the “availability” of exchange partners in solution. We believe that fast (ms) time points hold very valuable information and will be much more routinely accessed in the future, particularly for IDPs and other highly dynamic sequences. Accurate rate calibrations and salt considerations will be essential for studies of protein structure and dynamics in different buffers and with different ligands.

Another challenge for obtaining quantitative HDX-MS data is the unavoidable back-exchange, which is typically addressed by back-exchange corrections at the protein or peptide level. In proteins where the local environment of a residue changes due to quenching (denaturation) and digestion, the back-exchange rates are however not expected to correlate well with forward (and reverse) exchange prior to quenching since the higher-order structure is lost in the process and all residues become more or less deprotected. This means that the percentage of deuterium loss during back-exchange differs for each residue, which distorts experimental peptide uptake curves with respect to calculations. We avoid this problem by using disordered peptides without further digestion where the local environment of a residue remains the same after quenching with only the change in pH and deuterium content affecting all residues equally and in a predictable manner.

## Conclusions

HDX-MS has the potential ability to retrieve absolute structural information on a protein, in principle, at the resolution of the single amide–either experimentally^66^ or via an approach we described earlier^47^—which is critical to get a robust correlation between HDX data and atomistic models of protein structure and dynamics. To achieve this goal, it is essential to separate the effect of solution chemistry from the effects of sequence and structure on the HDX pattern and to use a correct, fully unstructured reference. Taken together, these considerations enable us to obtain an accurate and precise estimate of the intrinsic exchange rate *k*_*int*_.

The empirical predictions for the intrinsic exchange rate developed by the Englander Lab have proven useful since their first publication in 1993, but their validity has been questioned by several studies probing the HDX of intrinsically disordered proteins, with some observed rates exceeding the supposedly fastest possible rate based on the calibration with PDLA. We showed that these *k*_*int*_ calculations are more accurate when a trialanine peptide (3-Ala) is used as a reference instead of PDLA because the latter is not a completely unstructured peptide. To perform these calculations, we therefore suggest to use the rate constants for 3-Ala (reported in Table 1) in the Englander spreadsheet (https://hx2.med.upenn.edu/download.html) or, alternatively, to use our Python script available on GitHub (https://github.com/pacilab/exPfact).

The exchange kinetics of unstructured peptides are also a function of ionic strength. The presence of salt (NaCl) at 150 mM slows down the exchange, and therefore, the predictions mentioned above are not necessarily accurate when salt is present. In any case, the exchange predicted by the intrinsic rate should represent the fastest exchange possible for a given amino acid sequence at a given temperature and pH. We plan to further investigate such effects to determine a *salt correction factor*. Thus, we envisage in the future that a combined correction for temperature, pH, and salts will be possible, which will also allow us to define intrinsic rates for forward exchange more stringently as amide exchange rates of an individual amino acid within a given sequence, under standardized conditions of pH, temperature, and salt. This will serve to robustly deconvolve the true intrinsic rate of the covalent chemical structure, as determined by the protein sequence, from the extrinsic environmental conditions and from the effects of protein structural dynamics, the information that we ultimately want to reveal. A key element for the integration of protection factors with ensemble modeling and machine learning approaches is the availability of correctly calibrated HDX data, which requires the use of an “absolute” rather than “relative” reference such as the fully unstructured 3-Ala peptide.

## Supporting Information Available

Calculations showing that a minor change in pH can cause differences in the uptake curves that can be misclassified as significant; Circular dichroism (CD) spectra of the peptides in the peptide mixture; figures showing the distribution of standard deviations of the fractional uptake, and the effect of proline conformations on the H/D exchange of bradykinin; structural propensity of PDLA peptides of increasing lengths from molecular dynamics simulations; a table detailing the peptide mixture; a table summarizing the main conformations of bradykinin as determined by ion mobility-mass spectrometry (IM-MS); and additional references (PDF)
