# Supplementary material for: Recalibrating Protection Factors Using Millisecond Hydrogen/Deuterium Exchange Mass Spectrometry
Source: Anal Chem. 2025 Jan 29;97(5):2648–57. doi: 10.1021/acs.analchem.4c03631 (PMC11822740; doi:10.1021/acs.analchem.4c03631)
Supplement: Supplementary file 1 — ac4c03631_si_001.pdf [file ac4c03631_si_001.pdf]

Supplementary Information

Recalibrating Protection Factors Using Millisecond Hydrogen/Deuterium Exchange Mass Spectrometry

Michele Stofella[1], Neeleema Seetaloo[1,2,3], Alexander N. St John[1], Emanuele Paci[4*], Jonathan J. Phillips[2,3*] and Frank Sobott[1*]

[1]School of Molecular and Cellular Biology and Astbury Centre, University of Leeds, Leeds LS2 9JT, U. K.

[2]Living Systems Institute, University of Exeter, Exeter EX4 4QD, U. K.

[3]Department of Biosciences, University of Exeter, EX4 4QD, U. K.

[4]Dipartimento di Fisica e Astronomia, Università di Bologna, 40127 Bologna, Italy

*corresponding authors: e.paci@unibo.it, jj.phillips@exeter.ac.uk, f.sobott@leeds.ac.uk

Tale of contents

Supporting Figure 1. *Calculations showing that a minor change in pH can cause differences in the uptake curves that can be misclassified as significant……………..……………S-3*

Supporting Figure 2. *Circular dichroism (CD) spectra of the peptides in the peptide mixture…………………………………………………………………………….………S-4*

Supporting Figure 3. *Distribution of standard deviations of the fractional uptake…………..……S-5*

Supporting Figure 4. *The effect of proline conformations on the H/D exchange of bradykinin……………………………………………………………………………………........….S-6*

Supporting Figure 5. *Structural propensity of PDLA peptides of increasing lengths from Molecular Dynamics simulations……………………………………………..…………S-7*

Supporting Table 1. *Peptide mixture…………………………………………………………………S-9*

Supporting Table 2. *Main conformations of bradykinin as determined by ion mobility-mass spectrometry (IM-MS)……………………………………………………………………………….S-10*

References……………………………………………………………………………………….*S-11*

Supporting Figure 1. *Calculations showing that a minor change in pH can cause differences in the uptake curves that can be misclassified as significant.* We used Englander's intrinsic exchange rate calculations to determine the exchange rates of a completely unfolded poly-alanine peptide (sequence: AAAAAAAAAA) at temperature 300 K and at pH 7.0 (blue line) and pH 6.9 (orange line). The absolute uptake curves are calculated using Eq. 2 (without normalization). The difference between the two curves is evaluated and is found to be > 0.5 Da for timescales ranging from ~72 ms to ~360 ms.

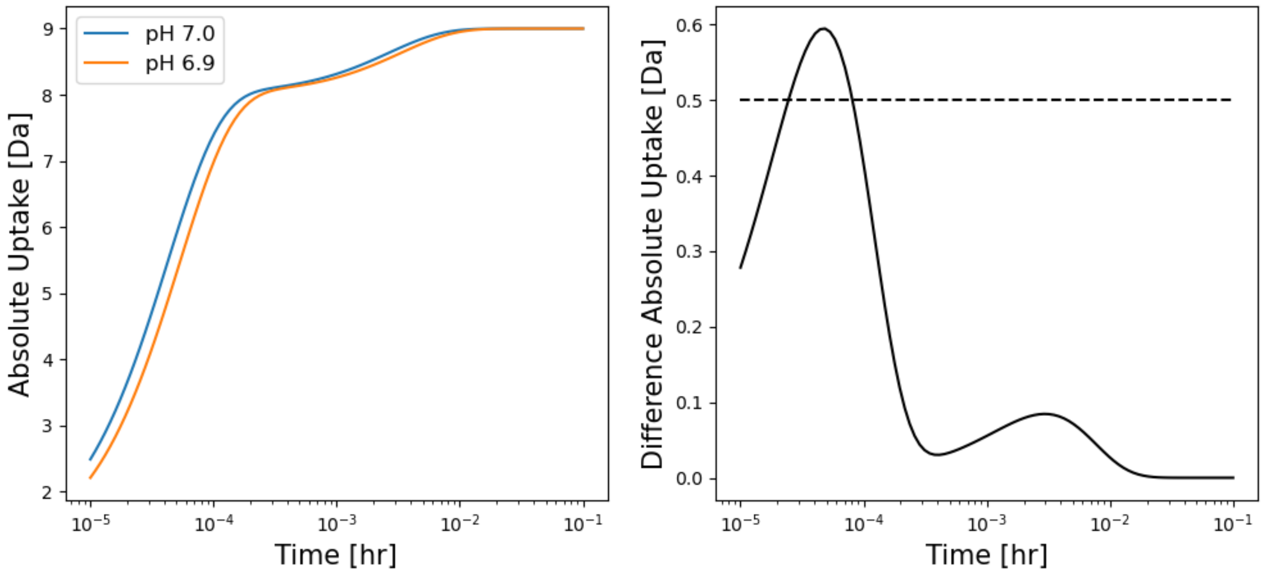

Supporting Figure 2. *Circular dichroism (CD) spectra of the peptides in the peptide mixture* (**Supporting Table 1**). CD experiments were performed on an Applied Photophysics Chirascan instrument (temperature 5°C), acquiring wavelengths in the range 180-250 nm. Wavelengths associated with voltages above 700 V were cropped as they are not reliable (there are not enough photons to have a statistically relevant measure). All spectra show a minimum at around 200 nm, which indicates that the primary behaviour of all peptides is that of a random coil. ANP does not have additional peaks, suggesting that it is the most unstructured peptide in the mixture (despite being the longest). The spectrum of bradykinin resembles poly-proline II in a conformation that "maximizes favourable interactions with the solvent"[1]. The spectrum of angiotensin has a second minimum at 218 nm which generally represents a beta-like structure; given the length of the angiotensin peptide (7 residues), this second peak most probably indicates a couple of residues with beta-like psi/phi angles preferences. It is not uncommon to find "conformational preferences" in unstructured peptides[2].

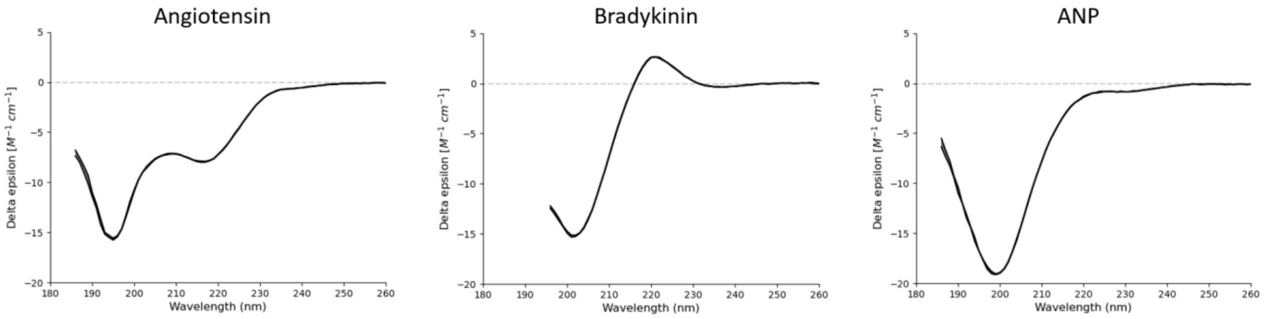

Supporting Figure 3. *Distribution of standard deviations of the fractional uptake* from the entire dataset, i.e. considering measurements from all replicates, time points and conditions. The red vertical dashed line represents the pooled standard deviation $\sigma_{pooled} = 0.041$.

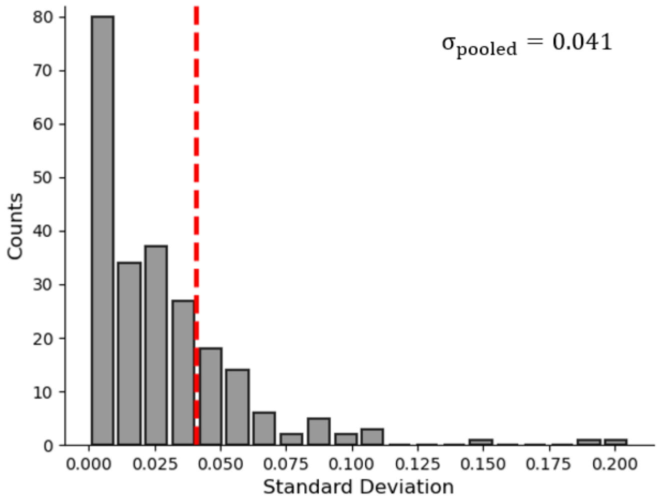

Supporting Figure 4. *The effect of proline conformations on the H/D exchange of bradykinin*. The fractional uptake calculated using 3-Ala as reference for different combinations of trans (P_T) or cis (P_C) prolines in the sequence of bradykinin. The table on the right summarizes the agreement with the experimental data shown in **Figure 1** via the sum of squared residuals (SSR). Independently of the conformation chosen, the SSR decreases when 3-Ala is used as reference instead of PDLA. The conformation depicted in **Figure 1** is highlighted in red.

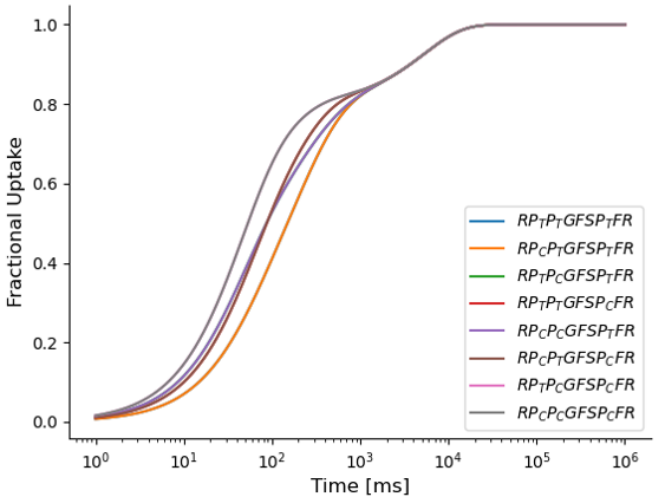

Sequence	SSR (3-Ala) SHOWN	SSR (PDLA) NOT SHOWN
$RP_TP_TGFSP_TFR$	0.107	0.245
**$RP_TP_TGFSP_CFR$**	**0.022**	**0.096**
$RP_TP_CGFSP_TFR$	0.027	0.100
$RP_CP_TGFSP_TFR$	0.107	0.244
$RP_CP_CGFSP_TFR$	0.027	0.100
$RP_CP_TGFSP_CFR$	0.022	0.096
$RP_TP_CGFSP_CFR$	0.008	0.023
$RP_CP_CGFSP_CFR$	0.008	0.023

Supporting Figure 5. *Structural propensity of PDLA peptides of increasing lengths from Molecular Dynamics simulations.* Snapshots were acquired every 100 ps of simulation and the secondary structural propensity of the peptide was calculated using the DSSP algorithm[3]. Average values are reported for helical propensity as a function of the amino acid index. Note that the residues at the first and last index corresponds to the acetyl and amide caps of the peptide.

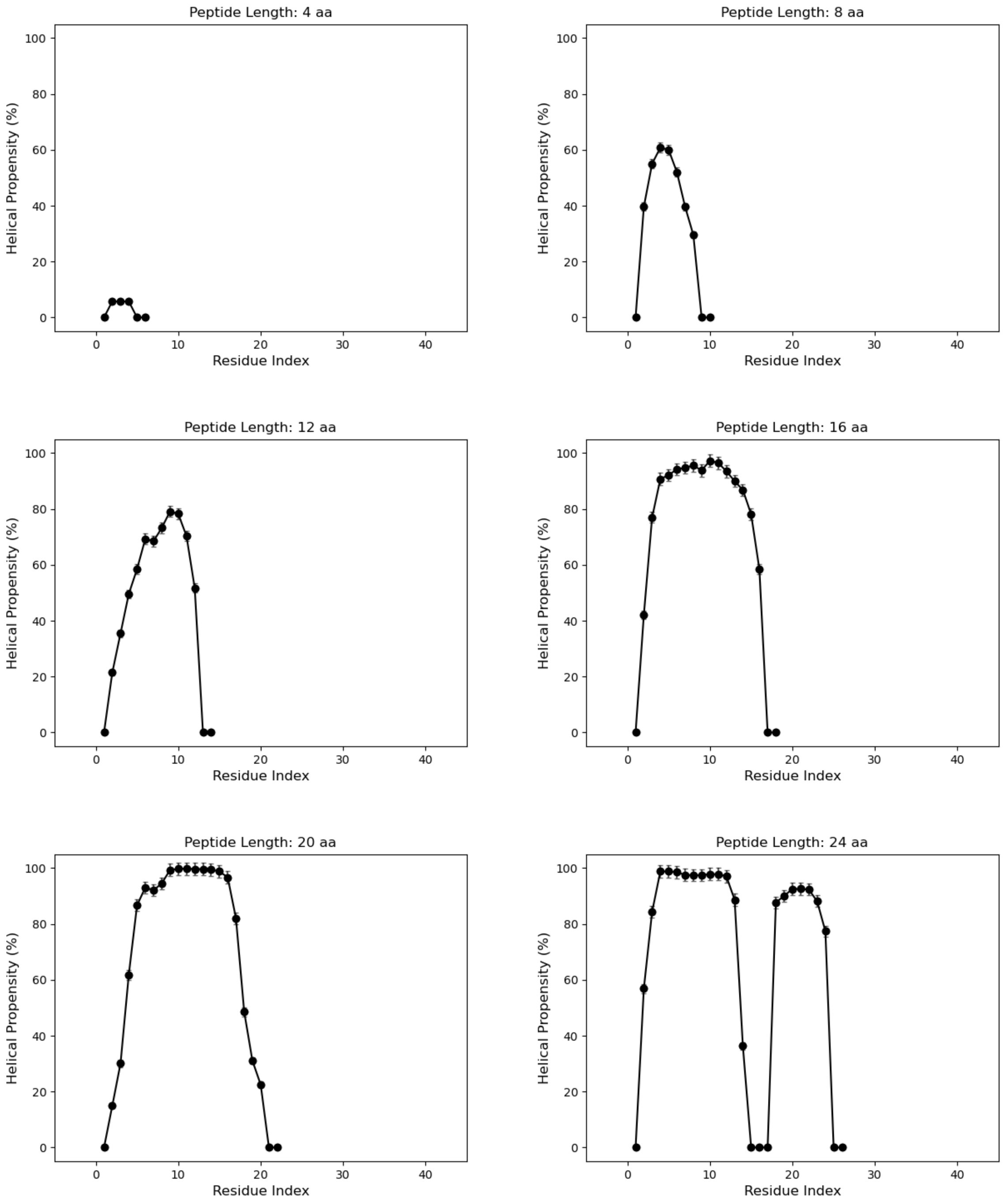

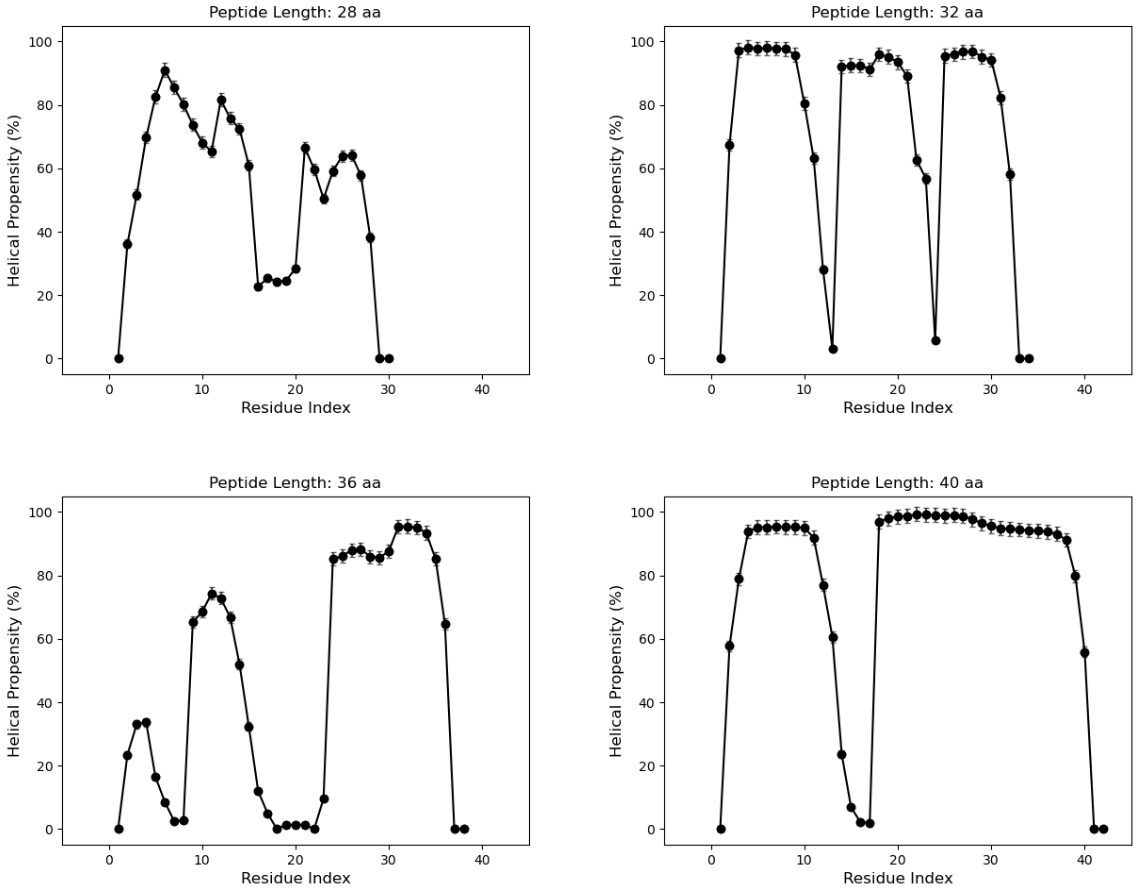
Peptide Length: 28 aa
Peptide Length: 32 aa
Peptide Length: 36 aa
Peptide Length: 40 aa
Helical Propensity (%)
Residue Index
0
20
40
60
80
100
0
10
20
30
40

Supporting Table 1. *Peptide mixture.* The peptide mixture was formed by three peptides: angiotensin, bradykinin and ANP 4-23 Amide rat. The sequences of the peptides and the measured monoisotopic masses are reported together with the Sigma Aldrich code. No information on the proline configuration of bradykinin is provided by the supplier. The monoisotopic mass detected for ANP (C-terminal amide) differs by -2 Da from the sequence mass (1595.75 Da), reflecting the formation of a disulfide bond between residues C4 and C15.

Peptide	Sequence	Experimental Monoisotopic Mass (Da)	Sigma Aldrich code
Angiotensin	DRVYIHP	889.455	A9202
Bradykinin	RPPGFSPFR	1059.550	90834
ANP	RSSCFGGRIDRIGAC - NH_2 [Cys4-Cys15]	1593.750	SCP0022

Supporting Table 2. *Main conformations of bradykinin as determined by ion mobility-mass spectrometry (IM-MS)*[4]. While it is not possible to derive the stereochemistry of prolines from HDX-MS data, the effect of prolines on neighbouring residues is a secondary yet important element to be considered when dealing with intrinsic exchange rate calculations (**Supporting Figure 4**). Although the *trans* configuration of prolines is usually considered to be more stable, the assumption of *cis*-prolines in unstructured peptides is not unrealistic[5]. Indeed, it was reported that "the cis-peptidyl-prolyl (cis-Pro) conformations in unfolded polypeptide chains are populated to significantly higher levels [with respect to folded proteins]"[6]. Using IM-MS, Pierson et al. have shown that multiple (up to 10) bradykinin conformers can coexist[7] with different combinations of cis/trans prolines[8]. Among the most abundant isomers, named A, B and C, the C state accounts for ~80% of the population[4]. The C conformation corresponds to the bradykinin isomer trans-Pro_2, trans-Pro_3, cis-Pro_7[8], which we assumed for the curve depicted in **Figure 1**. While a mixture of conformers is likely to exist in solution, it is realistic to assume that the HDX-MS data would capture the exchange of the most abundant conformer. Nevertheless, other conformations of bradykinin still provide good agreement with the experimental fractional uptake of bradykinin (**Supporting Figure 4**). Importantly, the use of 3-Ala as reference instead of PDLA improves the agreement with experimental data regardless of the bradykinin conformation considered.

**Bradykinin configuration**	**Pro_2**	**Pro_3**	**Pro_7**	**Abundance** (Quasi-Equilibrium)
A	*cis*	*cis*	*cis*	2 %
B	*cis*	*trans*	*trans*	16 %
C	*trans*	*trans*	*cis*	80 %

References

(1) Rucker, A. L.; Creamer, T. P. Polyproline II Helical Structure in Protein Unfolded States: Lysine Peptides Revisited. *Protein Sci* **2002**, *11* (4), 980–985. https://doi.org/10.1110/ps.4550102.

(2) Smith, L. J.; Fiebig, K. M.; Schwalbe, H.; Dobson, C. M. The Concept of a Random Coil: Residual Structure in Peptides and Denatured Proteins. *Folding and Design* **1996**, *1* (5), R95–R106. https://doi.org/10.1016/S1359-0278(96)00046-6.

(3) Kabsch, W.; Sander, C. Dictionary of Protein Secondary Structure: Pattern Recognition of Hydrogen-Bonded and Geometrical Features. *Biopolymers* **1983**, *22* (12), 2577–2637. https://doi.org/10.1002/bip.360221211.

(4) Pierson, N. A.; Valentine, S. J.; Clemmer, D. E. Evidence for a Quasi-Equilibrium Distribution of States for Bradykinin [M+3H]3+ Ions in the Gas Phase. *J Phys Chem B* **2010**, *114* (23), 7777–7783. https://doi.org/10.1021/jp102478k.

(5) Kienlein, M.; Zacharias, M.; Reif, M. M. Comprehensive Analysis of Coupled Proline Cis–Trans States in Bradykinin Using ωBP-REMD Simulations. *J. Chem. Theory Comput.* **2024**. https://doi.org/10.1021/acs.jctc.3c01356.

(6) Alderson, T. R.; Lee, J. H.; Charlier, C.; Ying, J.; Bax, A. Propensity for Cis-Proline Formation in Unfolded Proteins. *Chembiochem* **2018**, *19* (1), 37–42. https://doi.org/10.1002/cbic.201700548.

(7) Pierson, N. A.; Chen, L.; Valentine, S. J.; Russell, D. H.; Clemmer, D. E. Number of Solution States of Bradykinin from Ion Mobility and Mass Spectrometry Measurements. *J Am Chem Soc* **2011**, *133* (35), 13810–13813. https://doi.org/10.1021/ja203895j.

(8) Pierson, N. A.; Chen, L.; Russell, D. H.; Clemmer, D. E. Cis–Trans Isomerizations of Proline Residues Are Key to Bradykinin Conformations. *J Am Chem Soc* **2013**, *135* (8), 3186–3192. https://doi.org/10.1021/ja3114505.
